# Synthesis, Characterization, and Adhesion on Galvanized Steel of Original Thermoset Adhesive Films Based on Aza-Michael Addition Reaction

**DOI:** 10.3390/polym17131796

**Published:** 2025-06-27

**Authors:** Florian Cavodeau, Maurice Brogly, Jean-François Stumbe, Rémi Perrin

**Affiliations:** 1 Laboratoire de Photochimie et d’Ingénierie Macromoléculaires, Université de Haute Alsace, 3b Rue Alfred Werner, 68100 Mulhouse, France; florian.cavodeau@uha.fr (F.C.); jean-francois.stumbe@uha.fr (J.-F.S.); 2 Soprema, 15 Rue de Saint-Nazaire, 67100 Strasbourg, France; rperrin@soprema.fr

**Keywords:** thermoset adhesive, aza-Michael addition reaction, amine prepolymer, acrylate, adhesion

## Abstract

This study focuses first on the synthesis through an aza-Michael addition reaction of original linear diamine prepolymers and original amine/acrylate thermoset adhesives, and second on their thermal, mechanical and adhesion characterization. The major advantage of the aza-Michael addition reaction is that it takes place at room temperature, without a solvent and without a catalyst. Using the aza-Michael addition reaction, linear secondary diamine prepolymers were first synthesized with a control of the molecular weight, ranging from 867 to 1882 g mol^−1^. Then, aza-Michael reactions of diamine prepolymers with three different acrylates allowed the synthesis of new amine/acrylate thermoset adhesives. All the thermoset adhesives were characterized by rheology and thermal analysis, leading, once the crosslinking aza-Michael reaction had occurred, to soft thermoset networks with glass transition temperatures ranging from −23 to −8 °C, gel point times ranging from 40 min to 4 h, and a polar component of the surface energy ranging from 3 to 17 mJ m^−2^. Functionality of the acrylates directly influences the crosslinking rate, and a decreasing master curve is obtained when reporting crosslinking rate versus gel point time. Crosslinking density is controlled by the diamine prepolymer chain length. In a second step, thermoset adhesives were applied as thin films between two galvanized steel plates, and adhesion properties were evaluated through a lap-shear test. Results showed that the adhesive strength increases as the dynamic viscosity and molecular weight of the diamines prepolymer increases. Increasing the diamines prepolymer chain length results in an increase in strain at break, a decrease in the shear modulus, and a decrease in the maximum lap-shear strength. It is also observed that the adhesive strength decreases when the adhesive film thickness increases. Moreover, thermoset adhesives with high polarity and a surface energy similar to the surface energy of the substrate will favor high adhesion and a better adhesive strength of the assembly. Lastly, the nature of the acrylates and diamines prepolymer chain length allow tuning a wide range of adhesive strength and toughness of these original soft thermoset adhesives.

## 1. Introduction

The adhesives and sealants industries are in high demand for a new generation of environmentally friendly thermosetting adhesives that can be synthesized at room temperature without catalysts or solvents. In that way the aza-Michael reaction has gained some popularity regarding its use for the synthesis and formulation of crosslinked material. Indeed, multifunctional amines and acrylates are easily accessible; the synthesis generally does not require a catalyst or solvent and does not lead to the formation of byproducts [1,2]. Some studies include the use of the aza-Michael reaction for the preparation of dual-cured epoxy-acrylate-amine or silicon-based materials [3,4], but few only focus on pure aza-Michael thermoset materials [5,6]. In the present work, it was chosen to focus on amine/acrylate systems with a high reactivity, based on the aza-Michael addition [7,8]. The aza-Michael addition is a second-order reaction between a Michael acceptor, in this case an acrylate, and a Michael donor, which could be a primary or a secondary amine [9]. Its kinetics highly depends on the concentration of both Michael acceptor and donor. The reactivity of a secondary aliphatic amine is, however, stronger than a primary amine [10]. The main difference to consider is that the functionality of a primary amine with an acrylate is two and leads to the possibility to form bonds with two acrylates equivalent to one primary amine. The secondary amine obtained after the reaction between a first acrylate and a primary amine is sterically hindered and presents a drastically lowered reactivity. A new reaction could then be favored on another primary amine than on the hindered secondary one. Contrary to the synthesis of an amide, the amino ester function presents a secondary amine in the γ position from the carbonyl, which could also participate in the hindered function [11]. The amine in aza-Michael addition behaves both as the nucleophile and the base. The reaction does not need an additional base catalyst [12].

Furthermore, the reaction requires mild conditions to achieve high conversion and can easily take place at room temperature, contrary to more thermally activated curing reactions, as for epoxy systems. The absence of byproducts also implies the absence of volatiles and a 100% atom conservation during the crosslinking [13]. A last advantage that can be highlighted is that the aminoester bond obtained by aza-Michael addition could be hydrolyzed in low acidic conditions, opening a field of study on the recyclability of crosslinked polymers [14].

This work is dedicated to the synthesis, crosslinking characterization, and properties of original thermoset aza-Michael-based adhesives. Materials were obtained by means of the synthesis of prepolymers based on secondary amine functions obtained by aza-Michael addition between piperazine and a di-functional acrylate. The use of further multifunctional acrylates could then lead to a dense crosslinked network, presenting both softness and strength. The aim was also to produce amphiphilic molecules in order to favor the compatibility between the adhesive and a wide range of substrates.

In order to estimate the efficiency of the reaction at the interface, it was necessary to compare the properties of these adhesives with the ones obtained by stoichiometric mixture. The proof of concept was estimated using a standard lap-shear test on galvanized steel sheets.

## 2. Materials and Methods

### 2.1. Materials

Reactive secondary amine prepolymers were synthesized by the aza-Michael addition reaction of a di-functional cyclic secondary amine, piperazine (PIP; Molar weight = 86.14 g mol^−1^; supplied by BASF) and a di-functional linear acrylate 3-methyl-1,5-pentanediol diacrylate (MPDDA; Molar weight = 226.27 g mol^−1^; supplied by Sartomer). PIP was chosen because the reactivity of the amine functions is much higher for a cyclic amine compared to a linear one, which can accelerate the reaction with acrylate groups. The reactive secondary amines prepolymers obtained are named PIP-based prepolymers.

Crosslinked thermoset adhesives were synthesized by aza-Michael addition reaction of PIP-based prepolymers with three acrylates exhibiting an increase in their functionality of nearly 3 up to 4. They have been supplied by Sigma-Aldrich and are acrylate epoxidized soybean oil (AESO; Molar weight = 1124.12 g mol^−1^), trimethylolpropane triacrylate (TMPTA; Molar weight = 296.32 g mol^−1^), and di(trimethylolpropane) tetraacrylate (DTMPTTA; Molar weight = 466.52 g mol^−1^). The crosslinked thermoset adhesives obtained are named AESO/PIP-based prepolymers thermoset adhesives, TMPTA/PIP-based prepolymers thermoset adhesives and DTMPTTA/PIP-based prepolymer thermoset adhesives.

**Figure 1:**
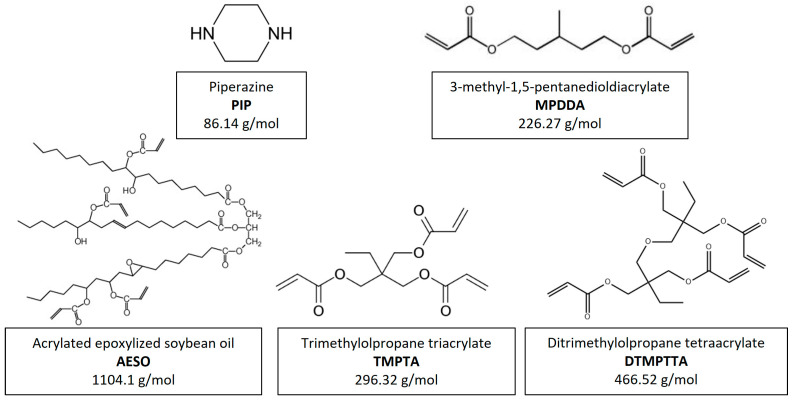
Representation of chemical formula of the amine and acrylate molecules.

Chemical formula of the amine and acrylates molecules used in the study are represented in Figure 1.

The liquids used for contact angle measurements, namely diiodomethane, 1-bromo-naphtalen and glycerol were supplied by Sigma-Aldrich. Deionized water was obtained using a Direct Q-3 water purification system (Merck-Millipore, Molsheim, France).

### 2.2. Synthesis of Linear Difunctional Secondary Amines Prepolymers

The molar weight and the number of repeat units can be controlled by using the Carothers equation and by adapting the initial proportions of PIP and MPDDA. This synthesis was realized in mass, without any solvent or catalyst, but needs to be heated in a round-bottom flask at 80 °C for 2 h to let PIP dissolve within the acrylate. PIP has been introduced in excess, leading to chain elongation and to the presence of PIP as terminal groups. The interest of the temperature is mostly to solubilize PIP, solid at room temperature, in the liquid MPDDA. The obtained prepolymers are expected to be liquid at room temperature, with a high viscosity.

In order to prepare prepolymers with different chain lengths and molar weights, MPDDA/PIP ratios were adapted based on Carothers equation, which expresses the degree of polymerization (DPn) as a function of the conversion rate of the reaction p.

(1) $$\mathrm{DPn}=\frac{1}{1\;-\;p},$$

PIP was introduced in excess in order to favor its presence as terminal group, and the stoichiometric ratio MPDDA/PIP, r, can be introduced in Carothers equation:

(2) $$\mathrm{DPn}=\frac{1+r}{1+r\;-\;2\mathrm{rp}},$$

In the case of a total conversion of the limiting reagent (p = 1), the obtained equation allows us to predict the DPn of the linear prepolymer as a function of the ratio r and, therefore, the stoichiometry of the reactive mixture. The reaction between PIP and MPDDA consists of a linear addition of two di-functional monomers with a repeating unit n corresponding to the succession of a PIP monomer and a MPDDA one. In this case DPn is equal to:

(3) $$DPn=\frac{1+r}{1-r}=2n,$$

The prediction of the molar weights of the synthetized prepolymers obtained by reaction between MPDDA and excess of PIP can then be performed and will be further confirmed by NMR analysis.

(4) $$\mathrm{Mn}=n\;\times \;\left({\mathrm{Mn}}_{\mathrm{PIP}}+{\mathrm{Mn}}_{\mathrm{MPDDA}}\right)+{\mathrm{Mn}}_{\mathrm{PIP}},$$

Four linear prepolymers were synthetized. Stoichiometric ratios, quantities introduced, predicted DPn, and predicted molar weights are presented in Table 1.

**Figure 2:**
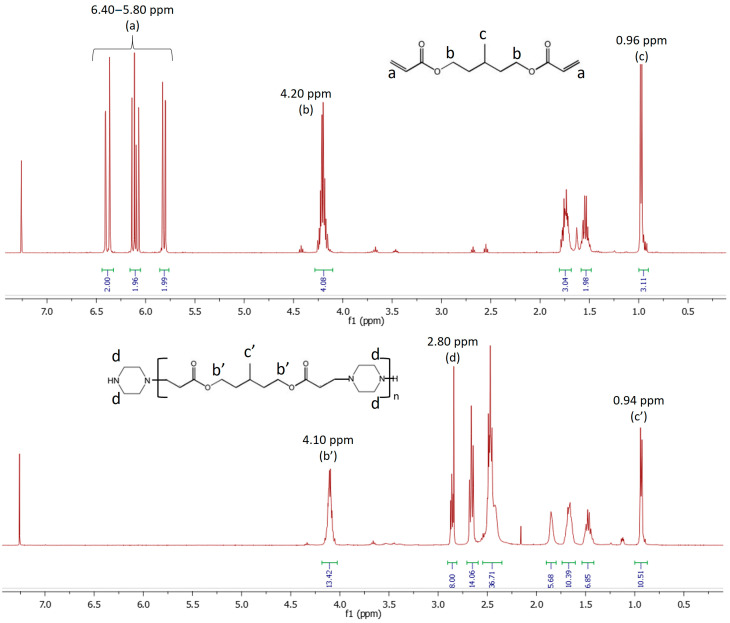
Principle of the ^1^H NMR characterization of PIP-based linear prepolymers (example of MPDDA/PIP-3/4 system).

Each synthesis was carried out for a total quantity of 20 to 25 g by mass, according to the ratios presented in Table 1. The products obtained were analyzed by DSC, rheometer, FTIR and ^1^H NMR to determine their structures and properties. In the case of ^1^H NMR analysis, characteristic protons are used to describe the structure of the prepolymers. The terminal groups of the linear molecules consist of PIP and have two CH_2_ groups in the α position of the terminal N-H. These protons are located at 2.80 ppm on the spectrum with an integration of 8 H, identical for each prepolymer. The second characteristic signal is located at 4.10 ppm and corresponds to the CH_2_ in the α position of the aliphatic O of the MPDDA part. Each repeating unit consists of one PIP and one MPDDA, corresponding to four equivalent H in the α position of the aliphatic O. The integration of the signal at 4.10 ppm allows to evaluate the number of PIP in the linear chain, and, therefore, the number of repeating units (n) in the prepolymers. This value was used to estimate the average molecular weight (Mn) value of the synthesized prepolymers according to Equation (4). The complete disappearance of the signals related to the acrylate groups (6.40–5.80 ppm) was used to guarantee the total reaction of these reactive functions [15]. The example of the MPDDA/PIP-3/4 prepolymer is presented in Figure 2. The signal at 4.10 ppm (b’) integrates for 13.4 protons when the signal at 2.80 ppm (d) is fixed at 8 protons and so corresponds to a “n” value equal to 3.35. According to Equation (4), the estimated average Mn is 1132 g mol^−1^.

Dynamic viscosities were measured at 25 °C and apparent glass transition temperatures (Tg) were determined by Differential Scanning Calorimetry (DSC) analysis. Obtained data are gathered in Table 2 and have been used for the formulation of adhesive systems. There is a difference between predicted and calculated Mn that can be explained by synthesis conditions leading to uncertainties. Some hypotheses for this difference could be a heterogeneity inside the reactive middle, an uncertainty due to the dissolution process of PIP within the acrylate, or a decrease in the kinetics due to the increase in viscosity when the chain length increases. Values are, however, considered to be similar.

### 2.3. Adhesive Systems

**Figure 3:**
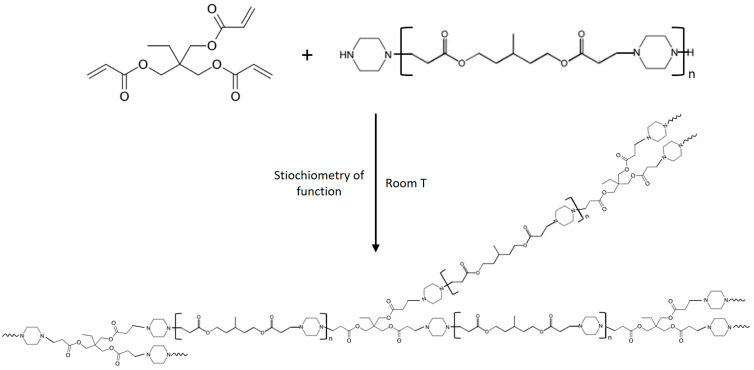
Schematic diagram of the crosslinking process between PIP-based prepolymers and a multifunctional acrylate (example of TMPTA).

The adhesive systems were made from the linear PIP-based prepolymers previously synthesized and commercial acrylates, namely AESO, TMPTA, and DTMPTTA, and involve aza-Michael addition to form the crosslinked network. As PIP-based prepolymers present only secondary amines as reactive functions, the stoichiometry and the kinetics of the reaction can be more controlled, without slowing down the reaction due to hindered secondary amine groups. The schematic crosslinking reaction is presented in Figure 3.

The objective is to compare the influence of acrylate functionality on the adhesive properties, in mass, at the interface and in the entire adhesive assembly. First, the adhesive systems were prepared by mixing each PIP-based prepolymer with an acrylate in a functionally stoichiometric mixture. All quantities, for each mixture, are listed in Table 3.

It was decided to carry out the mixing using a SpeedMixer DAC 150.1 FVZ (Hauschild, Hamm, Germany) apparatus. As aza-Michael addition starts when the amine and the acrylate are in contact, the mixing has to be fast and homogeneous enough to enable an optimized application on the substrate or for rheology analysis. A high mixing speed is also necessary to prepare homogeneous formulations with products with high viscosities, especially the PIP-based prepolymers and AESO. Since the acrylate content of AESO can vary between suppliers, the sample was analyzed by ^1^H NMR to estimate its functionality. Some NMR signals can be associated with constant chemical groups in AESO that do not vary in the presence of acrylate functionalities. Namely, the signal at 0.9 ppm corresponds to the terminal CH_3_ at the end of each triglyceride chain and has an integration of 9 H. The triglyceride group could also be identified with signals at 5.2 ppm (1 H) and 4.14–4.27 ppm (4 H) corresponding to the central CH and the two CH_2_ in α position, respectively. The CH_2_ signals in the α position of the carbonyl groups could also be identified at 2.3 ppm with an integration of 6 H. These were used as reference signals. The acrylate functions can then be analyzed with the three signals located between 5.8 and 6.4 ppm. In the case of the AESO used for the preparation of the adhesive, an integration of 2.7 H was found for each signal, indicating that there is an average of 2.7 acrylate functions for one AESO molecule. This is confirmed by the signal at 2.01 ppm relative to the CH of the linear chain linked to the acrylate group, with an integration of 2.7 H. The calculation for the preparation of the stoichiometric mixtures was carried out considering that the functionality of the AESO used for this study is 2.7 [16].

**Figure 4:**
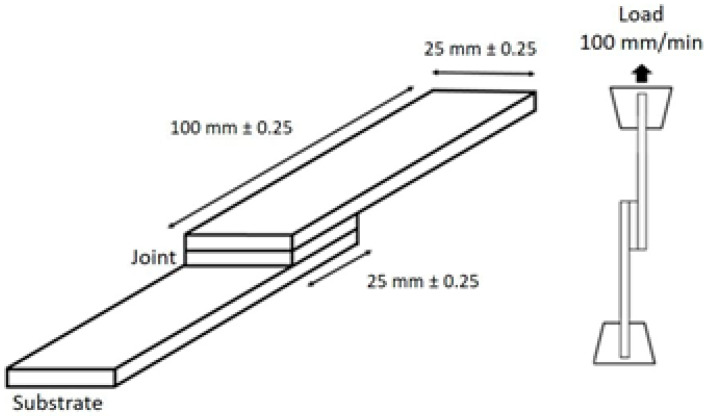
Principle and dimensions of the Single Lap-Shear specimen used for measuring adhesive strength.

The exact amount of each reagent was introduced into a suitable polypropylene container, for a total of 5 g of mixture. The reagents were then mixed for 3 min at a speed of 3000 rpm before direct application onto the steel substrates to be bonded. Approximately 1 g of the adhesive blended formulation was deposited on a 25 × 25 mm^2^ area at the edge of one steel plate, then the second steel plate was brought into contact to form the single-lap-shear adhesive assembly (Figure 4). To ensure contact between the two plates, clamps were used to hold the assembly for at least 2 h. After resting for 24 h at room temperature, the adhesive joint can be used for mechanical analyses without any post-curing step. The joint thickness was estimated using a digital micrometer with a resolution of 0.001 mm. First, the thickness of each steel plate was measured, then that of the assembly. The adhesive joint thickness is obtained by substracting the thickness of each steel plate from the thickness of the assembly. The thickness of the adhesive joint was estimated to be 0.05 mm. The adhesive mixture was also kept in the container during the curing process for further analyses. To conduct the single lap-shear experiments, two 25 × 100 mm^2^ galvanized steel plates with a thickness of 1.20 mm were chosen to be bonded together. These plates are conventionally used in the study of adhesion for the preparation of single-lap-shear specimens. Galvanized steel lap-shear specimens are used to limit the plastic deformation of the substrate and focus the experiment on the measurement of adhesive strength of the thermoset adhesive [17].

### 2.4. Methods Ans Apparatus

^1^H NMR analyses of the initial monomers and PIP-based prepolymers were performed in deuterated chloroform (CDCl_3_) at 20 °C on a Bruker 400 MHz spectrometer (Wissembourg, France).

The aza-Michael addition reaction between acrylates and linear secondary amine prepolymers was monitored by Fourier Transform InfraRed (FTIR) spectrometry in Attenuated Total Reflection (ATR) mode. Measurements were performed on the initial reactants and on the crosslinked thermoset adhesives with a Bruker Vertex 70 FTIR spectrometer (Wissembourg France) coupled to a Bruker Platinum ATR module mounted on a universal QuickLock device in the sample compartment. The ATR module is equipped with a diamond single-reflection crystal. The FTIR spectra were acquired from 4000 to 400 cm^−1^. The number of scans was fixed at 50 with a resolution of 4 cm^−1^. The reproducibility of the results was systematically checked. Spectral data were processed using the Bruker software system Opus 7.5. The conversion of double bonds in acrylates and terminal secondary amines was particularly closely monitored.

The reactivity of the adhesive systems was analyzed using an MCR 302 Rheometer (Anton Paar, Les Ulis, France) equipped with a parallel-plate mode to determine the gel point, the evolution of the G′ and G″ moduli, the dynamic viscosity (η), and the dephasing (δ). Measurements were carried out at a frequency of 1 Hz and a deflection angle of 0.2%. The rheological analyses were performed at 20 °C, and the test was stopped after 10 h.

Thermal properties were also analyzed by DSC using a Q200 DSC system (TA Instruments, Guyancourt, France) at a heating rate of 10 °C min^−1^ between −80 and 150 °C. Two cycles were performed to ensure complete conversion of the reactive functions of the crosslinked adhesives.

The adhesive properties of the systems are related to the intensity of intermolecular interactions between the adhesive and the substrate. Adhesion is considered optimal when the surface free energies (γ) and their polar, or non-dispersive (γ^ND^), and dispersive (γ^D^) components between the adherents are similar. These energies were estimated by contact angle measurement and the Owens–Wendt–Rabel and Kaelble (OWRK) calculation method, using distilled water, diiodomethane, 1-bromonaphthalene, and glycerol as reference liquids [18,19,20]. The polar component is considered to be the sum of polar, hydrogen, inductive and acid-base interactions. The sum of the polar and the dispersive components corresponds to the surface free energy.

(5) $$\gamma =\gamma^{D}+\gamma^{ND},$$

To determine γ of a surface, at least two liquids with known surface energy components (one mainly polar and one mainly dispersive) have to be used. In this work, a third was added to obtain more accurate results. Each measurement has been performed ten times on the steel substrates as well as on the crosslinked polymers used as adhesives. The components γ^D^ and γ^ND^ of each material is then calculated from the following equation.

(6) $$\sqrt{\gamma_{solid}^{D}}+\sqrt{\gamma_{solid}^{ND}}\sqrt{\frac{\gamma_{liquid}^{ND}}{\gamma_{liquid}^{D}}}=\frac{1}{2}\frac{\left[\gamma_{lquid}\left(1+\cos\theta \right)\right]}{\sqrt{\gamma_{liquid}^{D}}},$$

(7) $$x=\sqrt{\frac{\gamma_{liquid}^{ND}}{\gamma_{liquid}^{D}}};\;y=\frac{1}{2}\frac{\left[\gamma_{lquid}\left(1+\cos\theta \right)\right]}{\sqrt{\gamma_{liquid}^{D}}}$$

The graphical representation of y as a function of x allows the determination of the polar and dispersive components of the surface energy of a solid surface. The slope gives the polar component, and the vertical interception gives the dispersive component of the solid surface free energy. The adhesion of the adhesives to the steel substrates was estimated by a single lap-shear test using an M500-30AT tensile testing machine (Testometric, Rochdale, UK) at an elongation rate of 100 mm min^−1^ until complete fracture of the specimen. The adhesion area between the two substrates is 25 × 25 mm^2^. Depending on the tensile strength of the substrate, two load cells (50 and 3000 kg) were used. The measured characteristics are lap-shear strength (MPa), elongation at break (%), shear modulus (MPa), and toughness (N mm^−1^). Three adhesive assemblies were tested for each formulation. For the observation of fractures, an Olympus BX51 optical microscope (Olympus, Rungis, France) in reflection mode was used, associated with an Olympus DP20 CCD camera (Vision Engineering, Surrey, UK), with a magnification from ×50 to ×500.

## 3. Results

### 3.1. Amine/Acrylate Aza-Michael Addition Reactivity and Bulk Properties of the Adhesive

**Figure 5:**
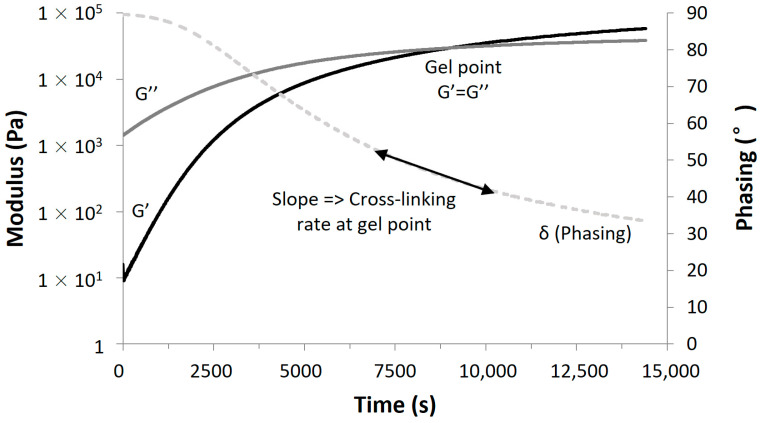
Parallel-plate rheology time curves (G′, G″ and δ) obtained at constant frequency for TMPTA/MPDDA/PIP-4/5 adhesive system.

The monitoring of the aza-Michael amine/acrylate addition reaction was performed using a parallel plate rheometer. After mixing the two components with the SpeedMixer, the uncured adhesive was deposited between the plates and analyzed at constant frequency and deflection angle until the gel point was reached (G′ = G″) corresponding to δ = 45°, and the experiment was stopped when G′ and G″ were linear and stable, indicating the end of the crosslinking reaction. The complex viscosity η* was also measured during the experiment to estimate its value when the gel point is reached. The curve δ = f(t) can be used to estimate the crosslinking rate. Specifically, the crosslinking rate at the gel point was estimated and expressed in s^−1^ to simplify the comparison between samples. Since the curve δ = f(t) is decreasing, the crosslinking speed was, therefore, calculated with the absolute value of the slope at the gel point [21]. The example of TMPTA/MDPPA/PIP-4/5 adhesive system measured at 20 °C is shown in Figure 5.

In Figure 5, the gel point was reached after around 4 h 30 min and the stabilization of G′ and G″ was not reached, even after 10 h of analysis. The crosslinking rate at gel point was 1.33 × 10^−3^ s^−1^ and the complex viscosity at gel point was 6070 Pa s. Characteristics of all the AESO/PIP-based prepolymers adhesives, measured at 20 °C, are gathered in Table 4, which also includes glass transition temperature measured by DSC (curves are presented as Supplementary Materials).

Table 5 and Table 6 gather parallel-plate rheology results obtained for, respectively, TMPTA/PIP-based prepolymers adhesives and DTMPTTA/PIP-based prepolymers adhesives, also measured at 20 °C.

For a given acrylate, the reactivity of adhesive systems increases as the PIP-based prepolymer chain length decreases, and the crosslinking rate at the gel point appears to be indirectly related to the gel point. A shorter secondary diamine reacts more rapidly with acrylate functional groups. Even if the number of reactive functional groups is equivalent in each mixture (for a given acrylate), the linear chain length of the PIP-based prepolymer results in a difference in reactivity. This observation seems logical because the polymerization rate strongly depends on the diffusion kinetics of the reactants toward each other, which is itself related to the chain length. Indeed, for PIP-based prepolymers, reactivity increases as Mn of prepolymers decreases, due to a decrease in viscosity as Mn decreases, inducing for a given acrylate a better diffusion between the PIP-based prepolymer and the acrylate, as confirmed by rheological measurements. A longer chain prepolymer exhibits higher dynamic viscosity, and when blended with an acrylate, rheological measurements show that the gel point is reached after a longer time and that the viscosity at the gel point is measured with a higher value, which also reflects the decrease in reactivity due to lower diffusion kinetics of the products.

The chain length of the PIP-based prepolymers also influences the crosslink density of the networks and their mechanical properties. Crosslink density is directly related to the glass transition (Tg) of the thermoset network. Indeed, as expected when comparing PIP-based prepolymers with a single acrylate, the Tg decreases with increasing chain length. All Tg values are negative, meaning that the thermoset adhesives obtained after crosslinking remain flexible at room temperature. This is an advantage in terms of the ability to dissipate energy for application in adhesive joints. The Tg of DTMPTTA/PIP-based adhesives is slightly higher than that of other systems due to a higher crosslink density, the monomer being tetrafunctional.

**Figure 6:**
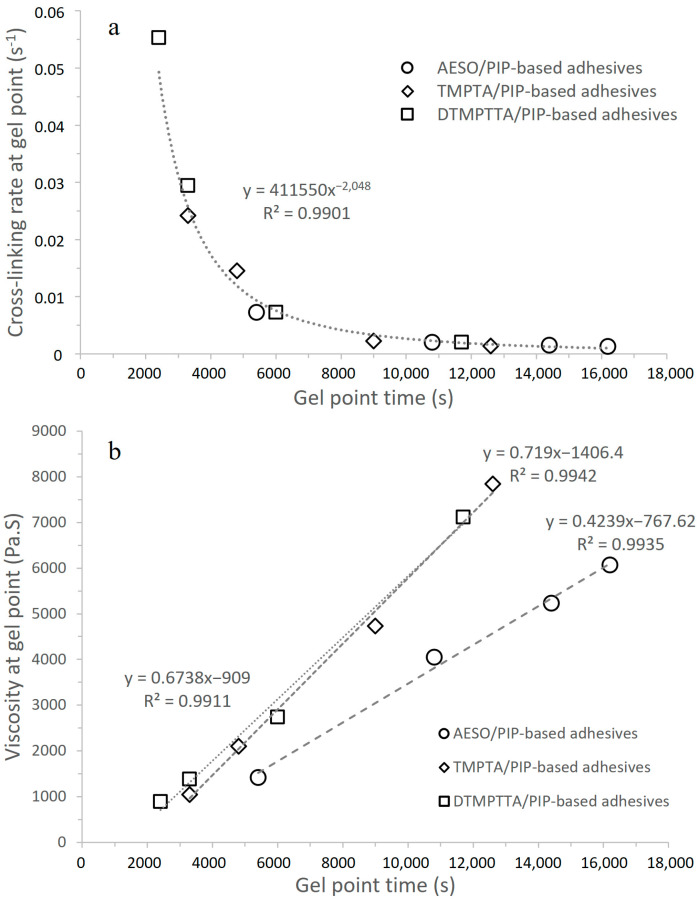
Crosslinking rate at gel point (**a**) and viscosity at gel point (**b**) as a function of gel point time for all the Acrylate/PIP-based prepolymers adhesive formulations.

In the case of aza-Michael addition, the reactivity strongly depends on the nature of the amine (primary, secondary, aromatic, etc.), which can influence the kinetics throughout the reaction [9]. For the synthesized PIP-based prepolymers, the nature of the amines can be considered equivalent (secondary amine). The reactivity of the system is then mainly related to the diffusion of the reactants between them and the availability of the reactive functions. This can be represented by comparing the crosslinking rates and the gel points. Indeed, one should expect that the longer the reaction takes to reach the gel point, the lower the crosslinking rate. All the systems presented previously follow a similar trend, as illustrated in Figure 6a.

A significant master curve is obtained. The nature and functionality of the acrylates directly influence the crosslinking rate. Tetrafunctional acrylate exhibits a more reactive function, which lowers the gel point and increases reactivity. For a given PIP-based prepolymer, a stoichiometric blend with DTMPTTA exhibits higher reactivity than TMPTA, and then AESO. However, reactivity is not strictly proportional to the functionality of the acrylate. A difference was observed by observing the change in viscosity at the gel point as represented in Figure 6b. As expected, viscosity is higher for longer gel time and is also related to the kinetics of the crosslinking reaction. Both TMPTA- and DTMPTTA-based adhesive formulations show a similar linear trend in complex viscosity evolution with gel time. In the case of AESO-based systems, however, the slope of the linear trend is lower, showing that at equivalent reaction times, the complex viscosity at the gel point is lower. This observation normally implies that diffusion of the reactants would be favored, which is not demonstrated by the kinetics since AESO-based systems have the longest gel time and the slowest crosslinking rate at the gel point. The structure of the AESO logically has an influence on the kinetics due to the hydrophobic chains, the nature of the acrylate functions, the size of the molecule, and its initial viscosity.

### 3.2. Surface Properties of the Aza-Michael Thermoset Adhesives

Kinetic of the crosslinking reaction has also a great influence on the required contact time of the Acrylate/PIP-based prepolymer adhesive formulations with a substrate to form an adhesive joint. After mixing, the adhesive formulations were cast into silicone molds. After crosslinking reaction has occurred, flexible thermoset adhesives were obtained for further physico-chemical analysis, including surface energy measurements.

Wettability measurements were performed on adhesive formulations. Results are presented in Table 7 that gather the contact angle values of droplets of reference liquids deposited on Acrylate/PIP-based prepolymers crosslinked thermoset adhesives, as well as the surface energies calculated using the Owens–Wendt method. γ_s_^ND^ and γ_s_^D^ are, respectively, the non-dispersive component and dispersive component of the surface energy, γ.

For a given acrylate, values of the surface energies and their non-dispersive and dispersive components are relatively close. The evolution of the polar and the dispersive components of the surface energy, however, follows a similar trend when the chain length of the PIP-based prepolymer increases. For the adhesives based on the same acrylate monomer, a decrease in the surface energy is observed when the chain length of the amine increases. Thermoset adhesives based on AESO as acrylate present a surface energy of 49 mJ m^−2^ when crosslinked with MPDDA/PIP-2/3 and decrease until 46 mJ m^−2^ when crosslinked with MPDDA/PIP-5/6. The decrease was measured from 49 mJ m^−2^ to 46 mJ m^−2^ with TMPTA and from 52 mJ m^−2^ to 47 mJ m^−2^ with DTMPTTA. However, standard deviations can bring uncertainties, as they may imply close values. Regarding dispersive and non-dispersive (polar) components of surface energies, a similar trend can be observed among each group of thermoset adhesives for a given acrylate. When the chain length of the amine prepolymer increases, the polar component of the surface energy decreases while the dispersive component increases. This observation is also confirmed by contact angle measurements, as the adhesive surface became more hydrophobic when the amine prepolymer chain length increased.

Table 8 shows the contact angle values of droplets of reference liquids deposited on galvanized steel substrate, as well as the surface energies calculated using the Owens–Wendt method.

Galvanized steel is considered as mainly hydrophilic due to the high value of the polar (non-dispersive) component of its surface energy.

Comparison of Table 7 and Table 8 results shows that all AESO-based and DTMPTA-based thermoset adhesives seem to be compatible with the galvanized steel surface, as their polar and dispersive components are close to each other [22,23,24]. TMPTA-based thermoset adhesives seem to be less compatible with the galvanized steel surface due to a lower polar component of the surface energy compared to galvanized steel. Contrary to what could have been expected, AESO does not seem to present the most hydrophobic behavior, even if it was synthesized from soybean oil, presenting three long carbonated chains. The presence of hydroxyls next to the acrylates and carbonyls in the glycerol part is supposed to increase the hydrophilic behavior of the adhesive surface. The absence of OH groups and the small size of TMPTA and DTMPTTA seem to favor the hydrophobic character induced by linear secondary amines, which also seems to be influenced by the chain length. Indeed, the hydrophobic character clearly increases with the chain length of the amine prepolymer.

## 4. Discussion

### Adhesive Properties of the Aza-Michael Thermoset Adhesives on Galvanized Steel

**Figure 7:**
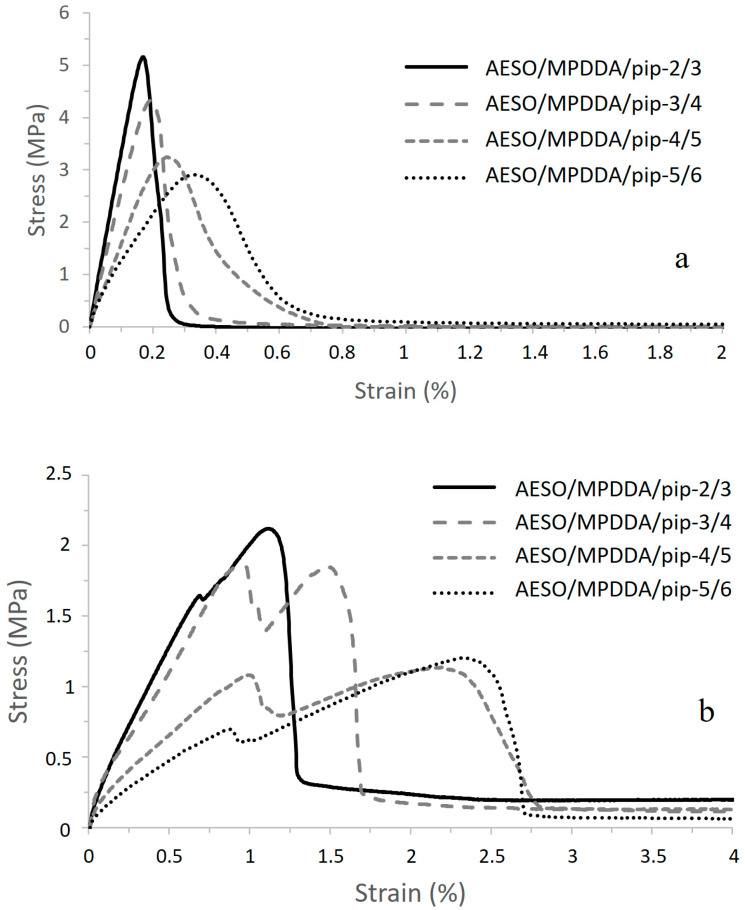
Single Lap-Shear test curves for Galvanized steel/AESO/PIP-based prepolymers thermoset adhesive assemblies (**a**) and with an adhesive joint thickness = 0.5 mm (**b**).

The aza-Michael adhesive formulations before crosslinking were applied to galvanized steel plates, and the adhesive assemblies were prepared as explained in Section 2.3. A weight of precisely 500 g was applied onto the assembly to ensure perfect contact between the adhesive and the substrates. After 24 h of rest at room temperature, the assemblies were tested according to the single lap-shear mechanical test at 100 mm/min until complete failure of the specimen. The curves obtained for the AESO/PIP-based prepolymer thermoset adhesives are shown in Figure 7a, and the measured characteristics are summarized in Table 9. The influence of the adhesive joint thickness on adhesive strength and mechanical resistance of the assembly was also investigated by preparing thicker joints between the galvanized steel plates. In this case, stoichiometric mixtures were realized, and the thickness of the adhesive was controlled using 0.5 mm Teflon wedges to prepare lap-shear specimens. Results obtained are presented in Figure 7b and Table 10.

**Figure 8:**
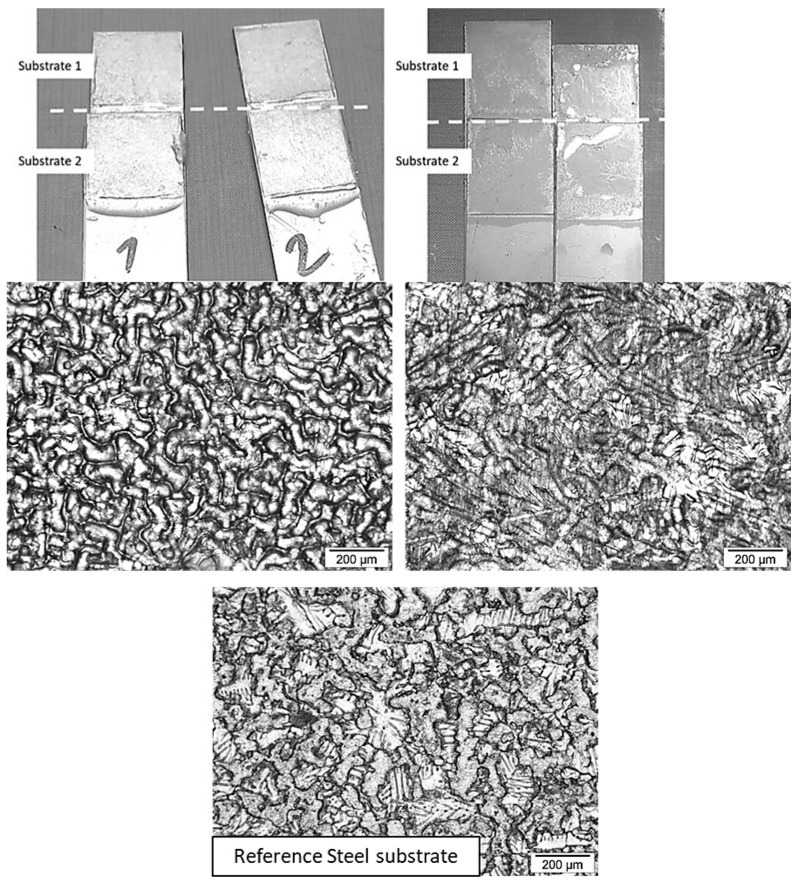
Single Lap-Shear assemblies after rupture for Galvanized steel/AESO/MPDDA/PIP-2/3 thermoset adhesive assembly (**left**) and Galvanized steel/AESO/MPDDA/PIP-5/6 thermoset adhesive assembly (**right**), with an adhesive joint thickness = 0.05 mm. Optical microscope images with a ×100 magnification.

Increasing the diamine prepolymer chain length influences the mechanical resistance of the assembly due to an increase in the molecular weight between the crosslinking points, inducing greater flexibility of the adhesive joint. This results in an increase in strain at break, a decrease in the shear modulus, and a decrease in the maximum lap-shear strength. Even though the strain at break increases with the diamine prepolymer chain length, the values remain very low, less than 1%, due to the high stiffness of the steel substrates. Since the toughness was calculated from the area under the stress vs. strain curves, it appears that a more flexible adhesive joint also has higher toughness and, therefore, better resistance to small deformations [25,26]. The thickness of the adhesive joint was 0.05 mm. In the case of AESO-based thermoset adhesives, the thickness was sufficient to cause cohesive failure within the joint, ensuring that the interface between the galvanized steel and the adhesive was greater than the strength of the adhesive itself. An example of failure is shown in Figure 8, comparing samples AESO/MPDDA/PIP-2/3 and AESO/MPDDA/PIP-5/6.

**Figure 9:**
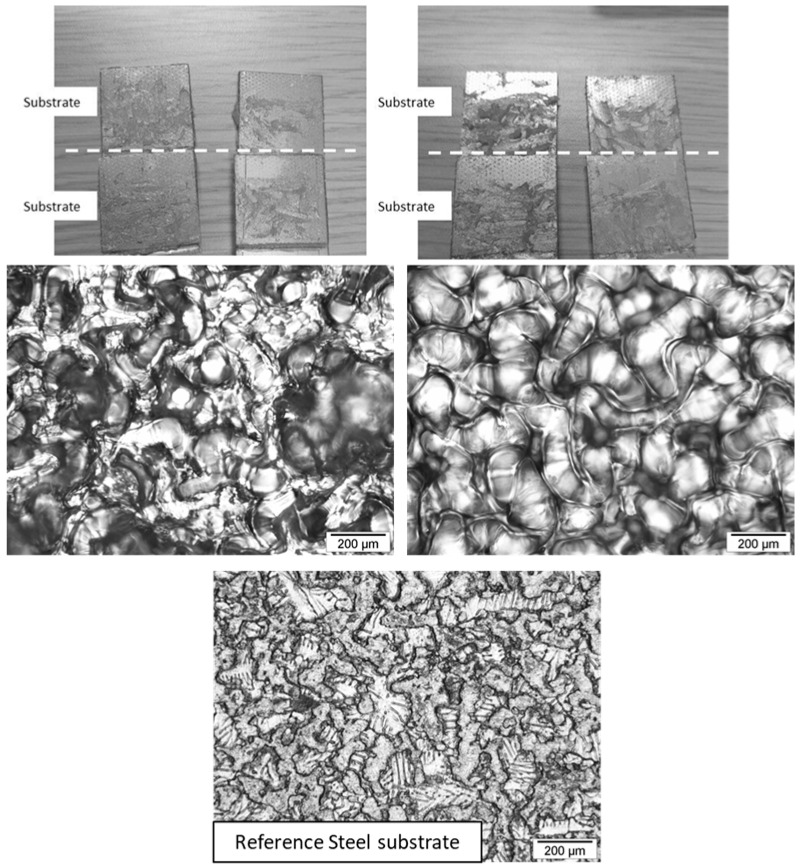
Single Lap-Shear assemblies after rupture for Galvanized steel/AESO/MPDDA/PIP-2/3 thermoset adhesive assembly (**left**) and Galvanized steel/AESO/MPDDA/PIP-5/6 thermoset adhesive assembly (**right**), with an adhesive joint thickness = 0.5 mm. Optical microscope images with a ×100 magnification.

As the adhesive joint presents a relative deformability compared to the substrate, the increase in its thickness also increases the global deformability of the assembly [27,28]. Indeed, the strain at break increases by a factor of ten when the adhesive joint thickness increases from 0.05 mm to 0.5 mm. This factor of ten also corresponds to the factor between the thicknesses of the two kinds of assemblies. The shear modulus decreases by a factor of 7 and the lap-shear strength by a factor of around 2.5. The fracture is still cohesive and goes through the adhesive joint layer with a crumbly aspect (Figure 9).

In this case, the interface always has a higher adhesive strength compared to the cohesion of the adhesive joint itself. It is also assumed that this thicker joint promotes the formation of an interphase and increases the adhesive strength of the interphase [29,30]. In the same time, the toughness and the strain at break of the assembly increase as the adhesive joint thickness does, due to a lower shear modulus and a higher ability to dissipate energy during the separation process. Then, in case of cohesive rupture, the adhesive strength is related to both the thickness of the joint and its own mechanical properties. A compromise could be found between the adhesive strength and deformability depending on the application [31].

**Figure 10:**
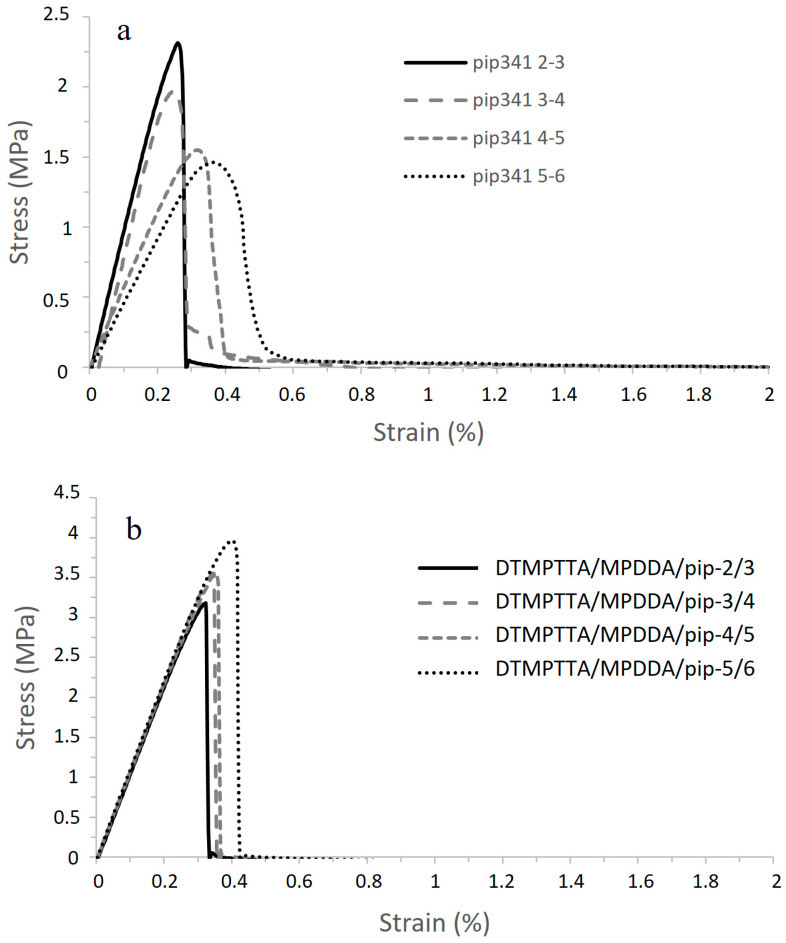
Single Lap-Shear test curves for Galvanized steel/TMPTA/PIP-based (**a**) and Galvanized steel/DTMPTTA/PIP-based (**b**) prepolymers thermoset adhesive assemblies with an adhesive joint thickness = 0.05 mm.

The same Single Lap-Shear experiments were conducted on TMPTA/PIP-based and DTMPTTA/PIP-based prepolymers thermoset adhesives. Results obtained are presented, respectively, in Figure 10a/Table 11, and in Figure 10b/Table 12.

**Figure 11:**
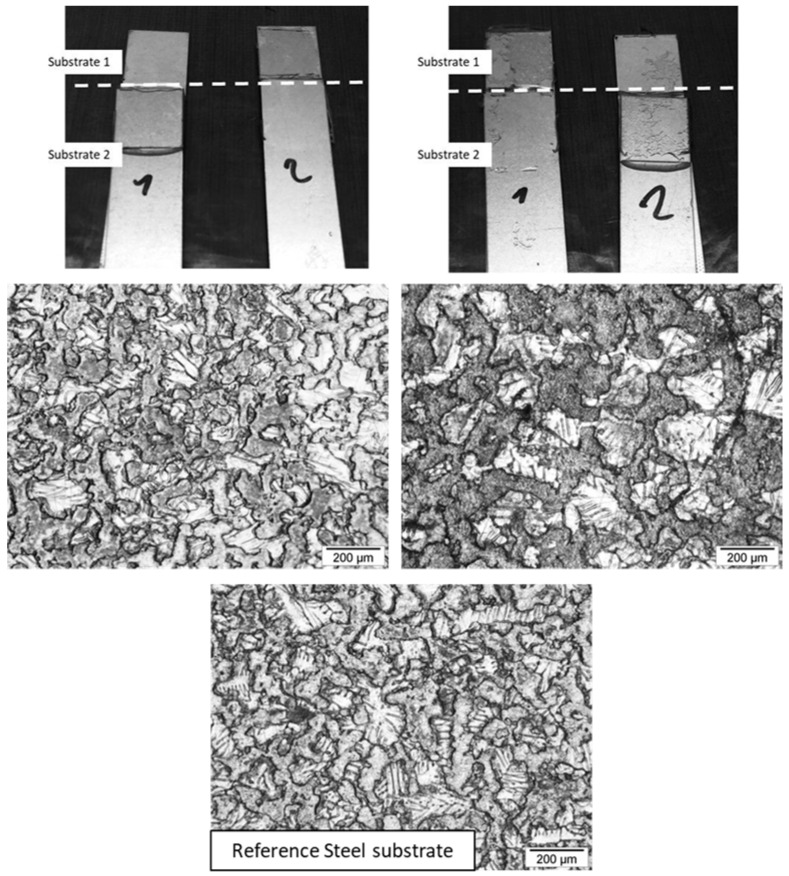
Single Lap-Shear assemblies after rupture for Galvanized steel/TMPTA/MPDDA/PIP-2/3 thermoset adhesive assembly (**left**) and Galvanized steel/TMPTA/MPDDA/PIP-5/6 thermoset adhesive assembly (**right**), with an adhesive joint thickness = 0.05 mm. Optical microscope images with a ×100 magnification.

Compared to AESO/PIP-based prepolymers thermoset adhesives, TMPTA/PIP-based prepolymers thermoset adhesives exhibit lower lap-shear strength and lower shear modulus, implying that these joints are less stiff and less resistant (Figure 10a and Table 11), inducing a slightly higher strain at break. Toughness values are somehow equivalent. Based on the structure of the acrylate used as a hardener, lower polymer chain mobility was expected, as the crosslinking node corresponds to a smaller molecule (TMPTA). DSC measurements on TMPTA/PIP-based prepolymer thermoset adhesives exhibit a negative Tg close to the Tg of AESO/PIP-based prepolymer thermoset adhesives, indicating a similar crosslink density, mainly influenced by the chain length of the linear diamine prepolymer. The mechanical properties of TMPTA/PIP-based prepolymer thermoset adhesive joints follow the same trend as AESO/PIP-based prepolymer thermoset adhesive. Lap-shear strength and shear modulus decrease with increasing chain length, while strain at break and toughness increase with increasing chain length. Weaker adhesive strength could then be explained by the nature of the locus of rupture. Indeed, TMPTA/PIP-based prepolymer thermoset adhesives always exhibit adhesive rupture (Figure 11). Indeed, TMPTA/PIP-based prepolymer thermoset adhesives exhibit very low non-dispersive (polar) components of the surface energy (Table 7) compared to AESO/PIP-based prepolymer thermoset adhesives. This contributes to decreasing the spreading ability of the adhesive formulation onto the galvanized steel substrate and to lowering the interfacial work of adhesion (W_a_). W_a_ is the work required to separate, reversibly, two phases in intimate molecular contact, and has been illustrated by various equations proposed by Young-Dupré (Equation (8)) and Kaelble (Equation (9)). “a” refers to the adhesive and “s” to the substrate. It is considered equivalent to the interfacial fracture energy without any energy dissipation.

(8) $$W_{a}=\gamma_{a}(1+\cos\theta ),$$

(9) $$W_{a}=2\sqrt{\left(\gamma_{a}^{D}\gamma_{S}^{D}\right)}+2\sqrt{\left(\gamma_{a}^{ND}\gamma_{S}^{ND}\right)},$$

The calculation of W_a_ for PIP-based adhesive systems using these equations, however, presents values not matching lap-shear test experiments. AESO-based adhesives show a Wa around 94–96 mJ m^−2^, TMPTA-based adhesives around 91–93 mJ m^−2^ and DTMPTTA-based adhesives around 98–100 mJ m^−2^. These values are very close, and DTMPTTA-based adhesives are supposed to present the higher interfacial fracture energy. Nevertheless, this prediction was not observed during the experiments [32]. The actual amount of work required to separate the two phases (i.e., adhesive strength) is, therefore, higher than estimated and likely involves other factors such as viscoelastic dissipation of the adhesive and/or plastic deformation of the substrate. Since AESO-based adhesives appear to be more deformable than the others, plastic dissipation could play a more important role and contribute more to adhesion, leading to a more cohesive fracture [33]. Moreover, the absence of OH groups and the small size of TMPTA seems to favor the hydrophobic character induced by PIP-based prepolymers, which also seems to be influenced by the chain length. Indeed, the hydrophobic character clearly increases with the chain length of the PIP-based prepolymers. All these effects contribute to weakening the interface, the adhesive strength, and, thus, inducing an adhesive rupture [34,35].

**Figure 12:**
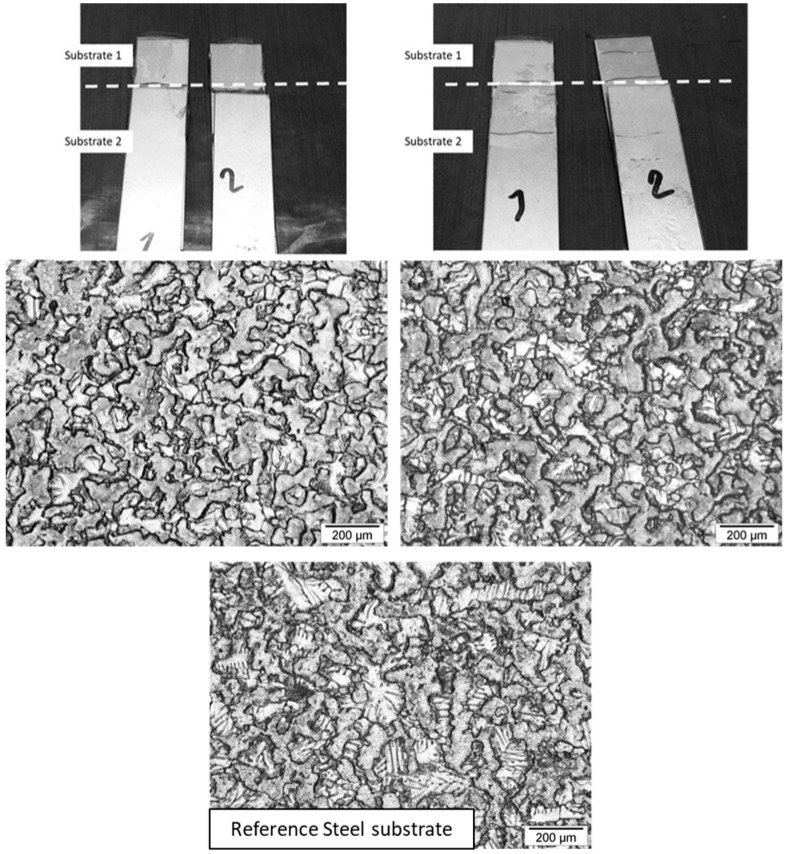
Single Lap-Shear assemblies after rupture for Galvanized steel/DTMPTTA/MPDDA/PIP-2/3 thermoset adhesive assembly (**left**) and Galvanized steel/DTMPTTA/MPDDA/PIP-5/6 thermoset adhesive assembly (**right**), with an adhesive joint thickness = 0.05 mm. Optical microscope images with a ×100 magnification.

Unlike the other systems, all DTMPTTA/PIP-based prepolymers thermoset adhesives exhibit a similar strain at break profile (Figure 10b and Table 12). This translates into a very similar shear modulus for each formulation, which appears independent of the PIP-based prepolymer chain length (approximately 1100 MPa). The strains at break increase with amine prepolymer chain length and are very similar to those observed for TMPTA/PIP-based prepolymers thermoset adhesives. They remain very low, reflecting the rigid aspect of the adhesive. The toughness increases from 46.3 N mm^−1^ to 74.3 N mm^−1^ with chain length and indicates a stronger adhesive, with a denser network induced by the four acrylate functions on DTMPTTA. Unlike AESO and TMPTA/PIP-based prepolymer thermoset adhesives, DTMPTTA/PIP-based prepolymer thermoset adhesives appear to exhibit a small increase in lap-shear strength with increasing PIP-based prepolymer chain length, indicating that a more resistant adhesive layer is formed and that chain length in this case influences only weakly joint deformability. Although DTMPTTA improves the joint mechanical properties and stiffness, the adhesive properties are weakened by the nature of the interface, similar to those observed for TMPTA/PIP-based prepolymers thermoset adhesives. The specimens after lap-shear tests do indeed exhibit adhesive rupture (Figure 12). This indicates that the strength of the interface remains lower than the cohesive energy of the adhesive joint [36]. The quality of the thermoset adhesive/galvanized steel substrate interface and their compatibility could explain this lower resistance of the interface. Indeed, it has been previously discussed that the absence of OH groups and the small size of DTMPTTA seems to favor the hydrophobic character induced by PIP-based prepolymers, which increases with chain length. Indeed, the hydrophobic character of the DTMPTTA/PIP-based prepolymers thermoset adhesives clearly increases with the chain length of the PIP-based prepolymers. This effect contributes to weakening the interface and, thus, induces an adhesive rupture.

**Figure 13:**
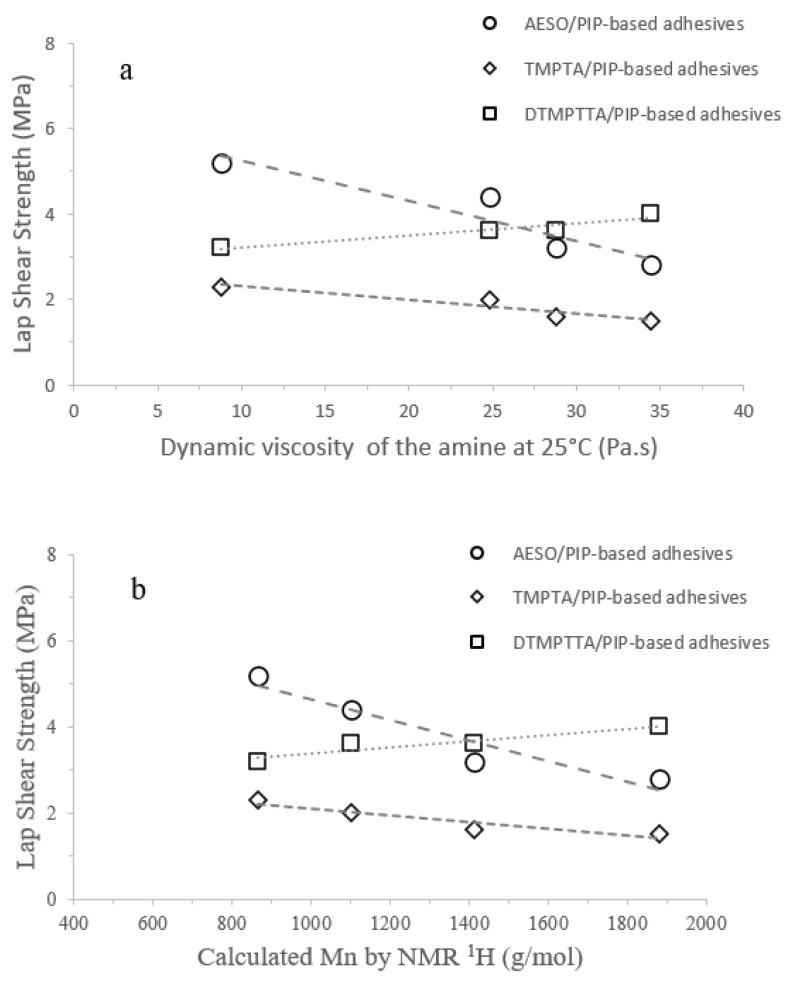
Influence of dynamic viscosities (**a**) of linear diamines and calculated Mn (**b**) on Lap-Shear strengths for all Acrylate/PIP-based prepolymers thermoset adhesives.

Figure 13 summarizes the link between lap-shear strengths of all experiments and properties of synthesized PIP-based prepolymers, such as dynamic viscosity and calculated Mn. The relationship between Mn and dynamic viscosity was already presented in Table 2. The dynamic viscosity increases with the chain length of the PIP-based prepolymers. AESO/PIP-based prepolymer and TMPTA/PIP-based prepolymer thermoset adhesives present a loss of lap-shear strength when the chain length of the PIP-based prepolymer decreases contrary to DTMPTTA/PIP-based prepolymer thermoset adhesives presenting a gain in lap-shear strength. This evolution leads to a trend inversion around 1400 g mol^−1^ (27 Pa s) where DTMPTTA/PIP-based prepolymer thermoset adhesives became more efficient than AESO/PIP-based prepolymer thermoset adhesive ones, considered as the most efficient formulation on galvanized steel for short chain lengths. TMPTA/PIP-based prepolymer thermoset adhesives remain less efficient. The linear decreasing trend could, however, imply that TMPTA/PIP-based prepolymer thermoset adhesives could present better adhesion than the AESO/PIP-based prepolymer thermoset adhesive for even higher Mn, with a crossing extrapolated around 2500–2600 g mol^−1^, corresponding to a dynamic viscosity around 38 Pa s [37]. Even if the chain length of the PIP-based prepolymers has a clear influence on adhesive properties, it should always be considered relative to the compatibility between the adhesive and the substrate [38].

To summarize and explain the impact of molecular weight (or chain length) of PIP-based prepolymers, nature of crosslink (controlled by acrylate functionality), and network structure on adhesion performance of thermoset adhesives, one has to consider that the adhesive strength (G) between a rigid substrate and an adhesive depends on two parameters, namely the thermodynamic work of adhesion (W_a_), and a viscoelastic dissipative function (ϕ(v,T)) related to the mechanical properties of the adhesive, separation speed (v), and temperature (T) [39]. In the specific case of a crosslinked adhesive [39], the adhesive strength depends also on M_c_, the average mass between crosslinks, according to Equation (10):

G = W_a_ × (1 + M_c_ × ϕ(v,T)) (10)

As a consequence, all the previous results could be explained by the fact that adhesive strength is tuned on one hand by the intensity of interfacial interactions (polar groups and OH groups in the case of AESO) through W_a_ and on the other hand by dissipative mechanisms that first increases as PIP-based prepolymers chain length does, and second, increases as crosslinking nods functionality does (2.7 for AESO, 3 for TMPTA and 4 for DTMPTTA).

## 5. Conclusions

Solventless and catalyst-free aza-Michael addition reaction allows linear secondary diamines prepolymers synthesis, at room temperature, with a control of the molecular weight, ranging from 867 to 1882 g mol^−1^. Then, aza-Michael reaction of these prepolymers with three different acrylates allow synthesis of original soft thermoset adhesives with glass transition temperatures ranging from −23 to −8 °C, as the prepolymer chain length decreases, and gel point times ranging from 40 min to 4 h. Functionality of the acrylates directly influences the crosslinking rate, and a decreasing master curve is obtained when reporting crosslinking rate versus gel point time. For a given amine/acrylate pair, a linear relationship was obtained between the complex viscosity at the gel point and the gel point time. Concerning surface characteristics, it is shown that these soft thermoset adhesives exhibit a range of polarity, having a polar component of the surface energy ranging from 3 to 17 mJ m^−2^. Their adhesion properties were investigated using a lap-shear test. Results showed that the adhesive strength increases as the dynamic viscosity and molecular weight of the diamine prepolymer increase. Increasing the diamine prepolymer chain length results in an increase in the strain at break, a decrease in the shear modulus, and a decrease in the maximum lap-shear strength. The most effective thermoset adhesive corresponds to the one whose polar and dispersive components of the surface energy are closest to the values determined for the galvanized steel substrate. Moreover, an increase in joint thickness reduces the adhesive strength but increases the strain at break and the toughness due to a higher deformability of the adhesive joint. Depending on the application, hydrothermal aging tests should also be considered in further work.

## Figures and Tables

**Table 3:** Weight percentages (wt%) of acrylate and amine used for the preparation of thermoset adhesive formulations at the stoichiometry of functions.

Acronym	MPDDA/PIP-2/3		MPDDA/PIP-3/4		MPDDA/PIP-4/5		MPDDA/PIP-5/6	
	Acrylate wt%	Amine wt%	Acrylate wt%	Amine wt%	Acrylate wt%	Amine wt%	Acrylate wt%	Amine wt%
AESO	48.5	51.5	42.6	57.4	36.6	63.4	30.3	69.7
TMPTA	18.6	81.4	15.2	84.8	12.2	87.8	9.5	90.5
DTMPTTA	16.9	83.1	13.8	86.2	11.1	88.9	8.6	91.4

**Table 1:** MPDDA/PIP ratios, weight percentages (wt%), predicted DPn and predicted Mn for the synthesis of linear secondary diamines prepolymers.

Acronym	MPDDA/PIP-2/3	MPDDA/PIP-3/4	MPDDA/PIP-4/5	MPDDA/PIP-5/6
MPDDA/PIP ratio	2/3	3/4	4/5	5/6
MPDDA wt%	65	68	70	72
PIP wt%	35	32	30	28
Predicted DPn	5	7	9	11
Predicted Mn (g mol^−1^)	711	1023	1336	1648

**Table 2:** Characteristics of the synthesized PIP-based linear prepolymers.

Acronym	MPDDA/PIP-2/3	MPDDA/PIP-3/4	MPDDA/PIP-4/5	MPDDA/PIP-5/6
Predicted Mn (g mol^−1^)	711	1023	1336	1648
Calculated Mn (g mol^−1^)	867	1132	1414	1882
Repeat units n	2.75	3.35	4.25	6.75
Tg (°C)	−43	−41	−39	−37
Dynamic viscosity at 25 °C (Pa s)	8.8	24.8	28.8	34.4

**Table 4:** Parallel-plate rheology results of AESO/PIP-based prepolymers adhesive formulations, at 1 Hz constant frequency and 20 °C.

	AESO MPDDA/PIP-2/3	AESO MPDDA/PIP-3/4	AESO MPDDA/PIP-4/5	AESO MPDDA/PIP-5/6
Gel point time (h)	1.5	3	4	4.5
End of reaction (h)	2.5	5	8.5	>10
η* at gel point (Pa s)	1420	4050	5230	6070
Crosslinking rate at gel point (s^−1^)	7.25 10^−3^	2.04 10^−3^	1.54 10^−3^	1.33 10^−3^
Tg (°C)	−16	−20	−22	−23

**Table 5:** Parallel-plate rheology results of TMPTA/PIP-based prepolymers adhesive formulations, at 1 Hz constant frequency and 20 °C.

	TMPTA MPDDA/PIP-2/3	TMPTA MPDDA/PIP-3/4	TMPTA MPDDA/PIP-4/5	TMPTA MPDDA/PIP-5/6
Gel point time (h)	0.916	1.333	2.5	4
End of reaction (h)	2	3.5	6	>10
η* at gel point (Pa s)	1050	2100	4730	7850
Crosslinking rate at gel point (s^−1^)	2.42 × 10^−2^	1.45 × 10^−2^	2.28 × 10^−3^	1.39 × 10^−3^
Tg (°C)	−15	−18	−20	−22

**Table 6:** Parallel-plate rheology results of DTMPTTA/PIP-based prepolymers adhesive formulations, at 1 Hz constant frequency and 20 °C.

	DTMPTTA MPDDA/PIP-2/3	DTMPTTA MPDDA/PIP-3/4	DTMPTTA MPDDA/PIP-4/5	DTMPTTA MPDDA/PIP-5/6
Gel point time (h)	0.666	0.916	1.666	3.25
End of reaction (h)	2	3	4.5	8
η* at gel point (Pa s)	890	1380	2740	7120
Crosslinking rate at gel point (s^−1^)	5.53 × 10^−2^	2.94 × 10^−2^	7.28 × 10^−3^	2.02 × 10^−3^
Tg (°C)	−8	−10	−13	−16

**Table 7:** Contact angle measurement of reference liquids and surface energy calculation by Owens–Wendt method on the Acrylate/PIP-based prepolymers crosslinked thermoset adhesives.

	Contact Angle (°)				Surface Energy (mJ m^−2^)		
	Water	Diiodomethane	1-Bromo-naphtalen	Glycerol	γ_s_^ND^	γ_s_^D^	γ
AESO/MPDDA/PIP-2/3	58 ± 2	40 ± 3	42 ± 2	39 ± 2	17 ± 2	32 ± 2	49 ± 2
AESO/MPDDA/PIP-3/4	60 ± 1	38 ± 3	42 ± 3	40 ± 1	15 ± 2	33 ± 2	47 ± 3
AESO/MPDDA/PIP-4/5	65 ± 4	40 ± 3	39 ± 2	44 ± 3	12 ± 3	34 ± 3	46 ± 3
AESO/MPDDA/PIP-5/6	67 ± 3	42 ± 2	28 ± 2	50 ± 4	10 ± 3	36 ± 3	46 ± 3
TMPTA/MPDDA/PIP-2/3	70 ± 1	33 ± 1	18 ± 2	42 ± 2	6 ± 1	41 ± 1	47 ± 1
TMPTA/MPDDA/PIP-3/4	70 ± 2	30 ± 2	15 ± 3	40 ± 1	5 ± 2	42 ± 2	47 ± 2
TMPTA/MPDDA/PIP-4/5	78 ± 3	28 ± 2	12 ± 2	45 ± 2	4 ± 2	44 ± 2	48 ± 2
TMPTA/MPDDA/PIP-4/6	84 ± 4	37 ± 3	17 ± 3	46 ± 1	3 ± 3	43 ± 3	46 ± 3
DTMPTTA/MPDDA/PIP-2/3	60 ± 1	28 ± 2	25 ± 2	41 ± 2	13 ± 2	39 ± 2	52 ± 2
DTMPTTA/MPDDA/PIP-3/4	62 ± 3	17 ± 3	19 ± 2	44 ± 3	11 ± 3	41 ± 3	52 ± 3
DTMPTTA/MPDDA/PIP-4/5	69 ± 3	22 ± 3	24 ± 2	46 ± 2	10 ± 2	41 ± 2	51 ± 2
DTMPTTA/MPDDA/PIP-5/6	76 ± 2	26 ± 2	20 ± 1	52 ± 3	9 ± 2	42 ± 2	51 ± 2

**Table 8:** Contact angle measurement of reference liquids and surface energy calculation by the Owens–Wendt method on the galvanized steel substrate.

	Contact Angle (°)				Surface Energy (mJ m^−2^)		
	Water	Diiodomethane	1-Bromo-naphtalen	Glycerol	γ_s_^ND^	γ_s_^D^	γ
Galvanized steel	62 ± 1	43 ± 2	21 ± 2	45 ± 3	12 ± 2	36 ± 2	48 ± 2

**Table 9:** Single Lap-Shear test results for Galvanized steel/AESO/PIP-based prepolymers thermoset adhesive assemblies.

	Lap-Shear Strength (MPa)	Strain at Break (%)	Shear Modulus (MPa)	Toughness (N mm^−1^)
AESO/MPDDA/PIP-2/3	5.2 ± 0.4	0.16 ± 0.04	3330 ± 64	32.4 ± 0.8
AESO/MPDDA/PIP-3/4	4.4 ± 0.2	0.20 ± 0.02	2620 ± 80	31.2 ± 0.5
AESO/MPDDA/PIP-4/5	3.2 ± 0.2	0.25 ± 0.02	2020 ± 11	40 ± 3
AESO/MPDDA/PIP-5/6	2.8 ± 0.3	0.35 ± 0.01	1050 ± 25	53.2 ± 0.6

**Table 10:** Single Lap-Shear test results for Galvanized steel/AESO/PIP-based prepolymers thermoset adhesive assemblies with an adhesive joint thickness = 0.5 mm.

	Lap-Shear Strength (MPa)	Strain at Break (%)	Shear Modulus (MPa)	Toughness (N mm^−1^)
AESO/MPDDA/PIP-2/3	2.1 ± 0.2	1.14 ± 0.04	480 ± 5	141 ± 4
AESO/MPDDA/PIP-3/4	1.8 ± 0.3	1.49 ± 0.06	400 ± 15	173 ± 6
AESO/MPDDA/PIP-4/5	1.1 ± 0.2	2.16 ± 0.06	290 ± 12	185 ± 9
AESO/MPDDA/PIP-5/6	1.2 ± 0.1	2.36 ± 0.04	175 ± 5	220 ± 10

**Table 11:** Single Lap-Shear test results for Galvanized steel/TMPTA/PIP-based prepolymers thermoset adhesive assemblies with an adhesive joint thickness = 0.05 mm.

	Lap-Shear Strength (MPa)	Strain at Break (%)	Shear Modulus (MPa)	Toughness (N mm^−1^)
TMPTA/MPDDA/PIP-2/3	2.3 ± 0.2	0.26 ± 0.02	1015 ± 23	29.7 ± 0.8
TMPTA/MPDDA/PIP-3/4	2.0 ± 0.3	0.25 ± 0.04	1050 ± 12	30.6 ± 0.6
TMPTA/MPDDA/PIP-4/5	1.6 ± 0.2	0.33 ± 0.03	590 ± 17	33.0 ± 0.2
TMPTA/MPDDA/PIP-5/6	1.5 ± 0.1	0.38 ± 0.02	490 ± 16	37.3 ± 0.3

**Table 12:** Single Lap-Shear test results for Galvanized steel/DTMPTTA/PIP-based prepolymers thermoset adhesive assemblies with an adhesive joint thickness = 0.05 mm.

	Lap-Shear Strength (MPa)	Strain at Break (%)	Shear Modulus (MPa)	Toughness (N mm^−1^)
DTMPTTA/MPDDA/PIP-2/3	3.2 ± 0.2	0.32 ± 0.02	1090 ± 16	46.3 ± 0.2
DTMPTTA/MPDDA/PIP-3/4	3.6 ± 0.4	0.34 ± 0.03	1095 ± 10	56.4 ± 0.3
DTMPTTA/MPDDA/PIP-4/5	3.6 ± 0.3	0.35 ± 0.01	1120 ± 28	59.5 ± 0.3
DTMPTTA/MPDDA/PIP-5/6	4.0 ± 0.3	0.41 ± 0.02	1135 ± 12	74.3 ± 0.2

## Data Availability

The raw data supporting the conclusions of this article will be made available by the authors upon request.

## Supplementary Materials

The following supporting information can be downloaded at: https://www.mdpi.com/article/10.3390/polym17131796/s1. DSC Curves of PIP-based prepolymers and all Acrylates/PIP-based prepolymers thermoset adhesives. Supplementary S1: PIP-based prepolymers; Supplementary S2: AESO/PIP-based prepolymers thermoset adhesives; Supplementary S3: TMPTA/PIP-based prepolymers thermoset adhesives; Supplementary S4: DTMPTTA/PIP-based prepolymers thermoset adhesives.

## Abbreviations

The following abbreviations are used in this manuscript:

PIP	PIPerazine
MPDDA	3-Methyl-1,5-PentaneDiol DiAcrylate
AESO	Acrylate Epoxidized Soybean Oil
TMPTA	TriMethylolPropane TriAcrylate
DTMPTTA	Di(TriMethylolPropane) TeTraAcrylate
