# Supplementary figures and images for: Synthesis, Characterization, and Adhesion on Galvanized Steel of Original Thermoset Adhesive Films Based on Aza-Michael Addition Reaction
Source: Polymers (Basel). 2025 Jun 27;17(13):1796. doi: 10.3390/polym17131796 (PMC12251997; doi:10.3390/polym17131796)

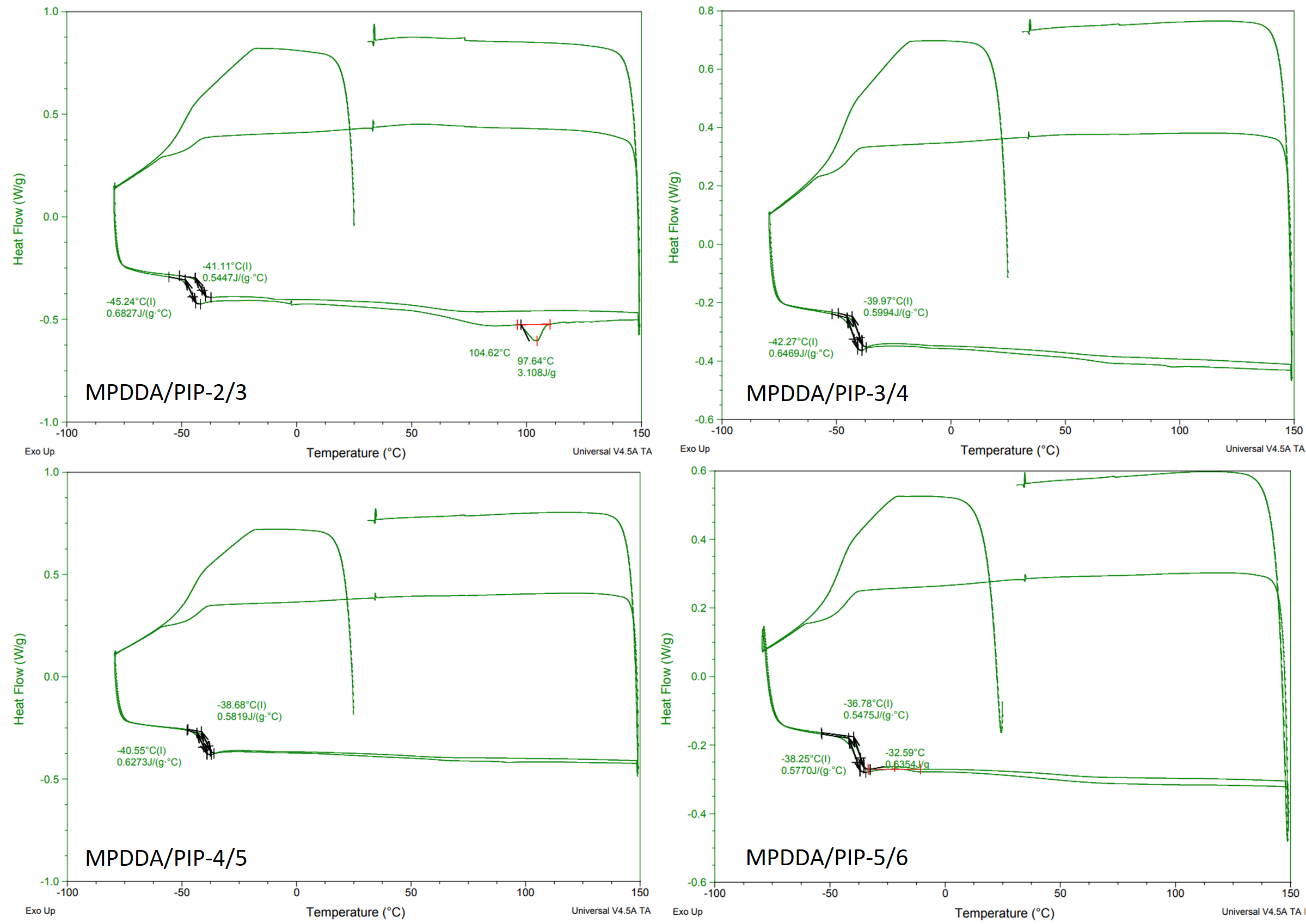

Supplement: Supplementary file 1 [file polymers-17-01796-s001.zip › Supplementary S2 v2.bmp]

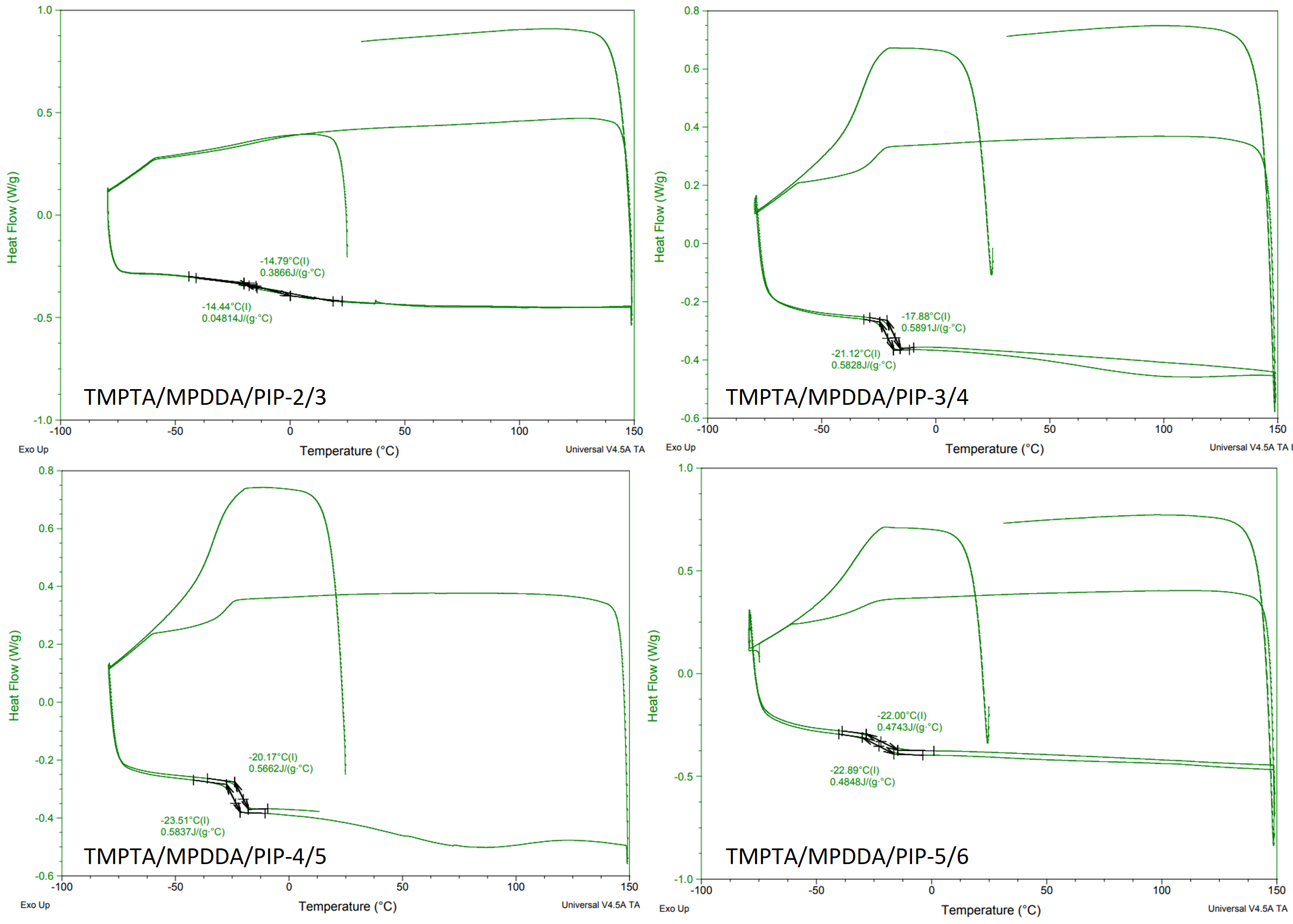

Supplement: Supplementary file 1 [file polymers-17-01796-s001.zip › Supplementary S3 v2.bmp]

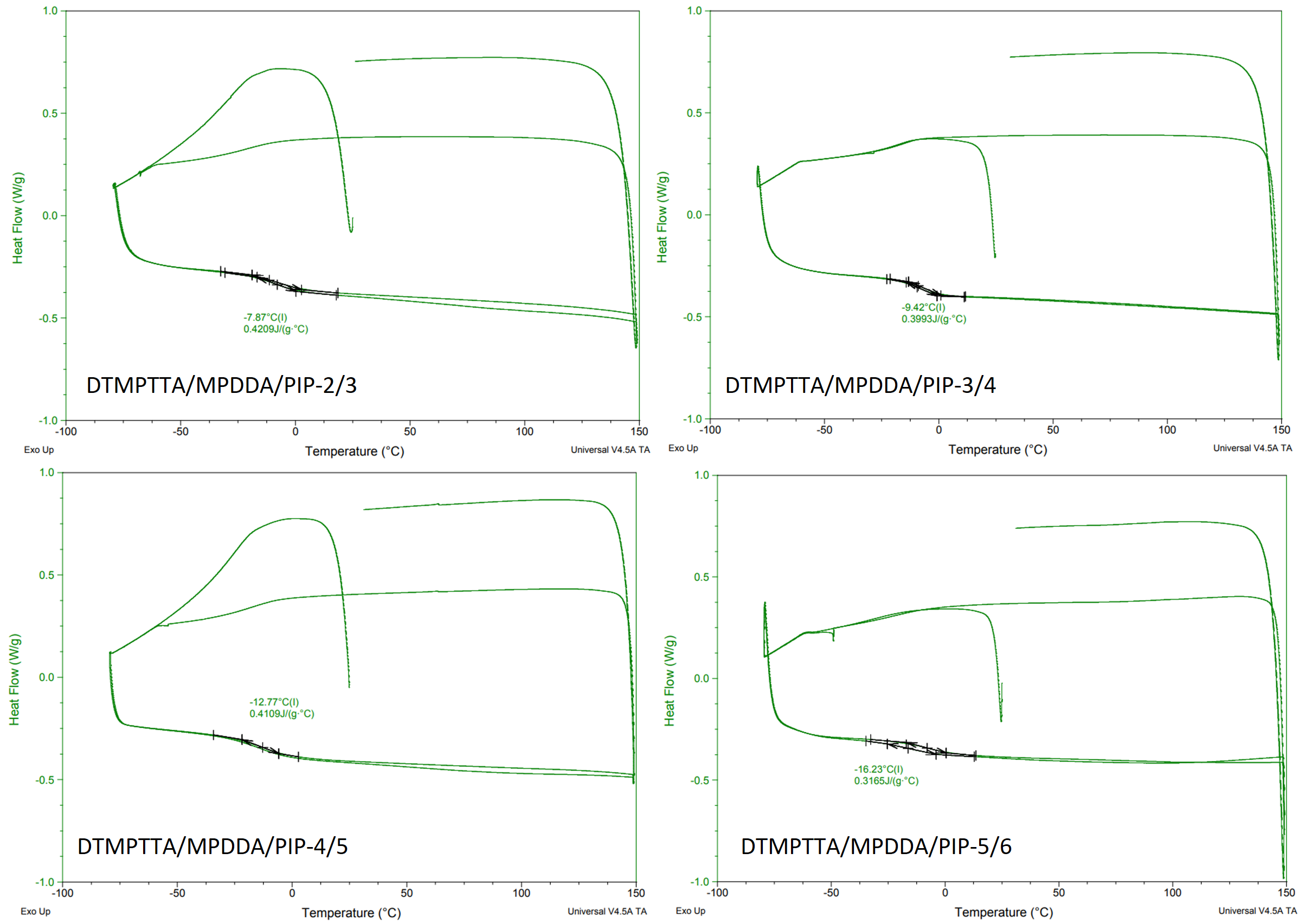

Supplement: Supplementary file 1 [file polymers-17-01796-s001.zip › Supplementary S4 v2.bmp]

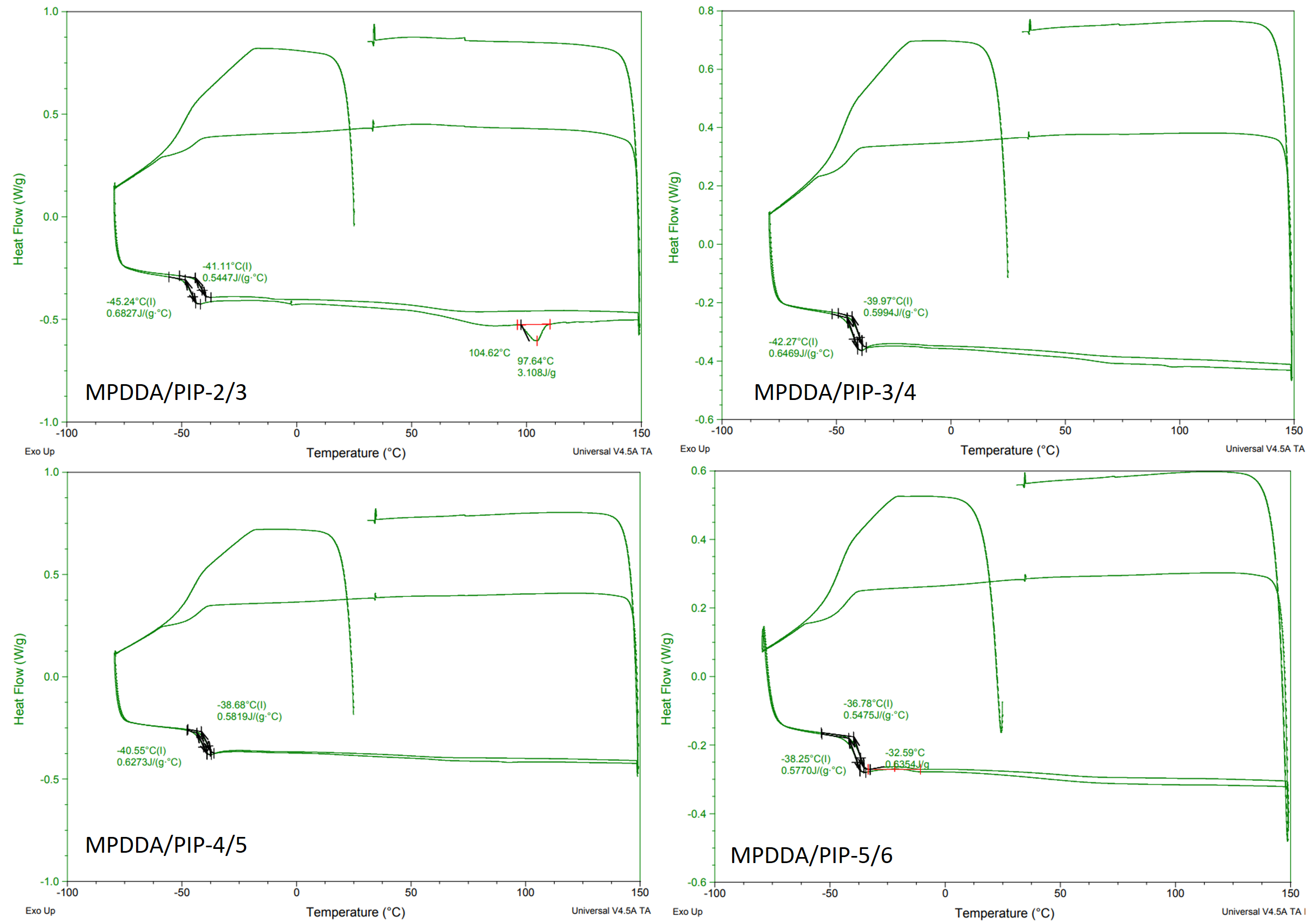

Supplement: Supplementary file 1 [file polymers-17-01796-s001.zip › Supplementary S1 v2.bmp]
